# Accuracy and reproducibility of virtual cutting guides and 3D-navigation for osteotomies of the mandible and maxilla

**DOI:** 10.1371/journal.pone.0173111

**Published:** 2017-03-01

**Authors:** Jonathan M. Bernstein, Michael J. Daly, Harley Chan, Jimmy Qiu, David Goldstein, Nidal Muhanna, John R. de Almeida, Jonathan C. Irish

**Affiliations:** 1 Guided Therapeutics (GTx) Program, TECHNA Institute, University of Toronto, Princess Margaret Cancer Centre, Toronto, Ontario, Canada; 2 Department of Otolaryngology - Head & Neck Surgery / Surgical Oncology, University of Toronto, Princess Margaret Cancer Centre, Toronto, Ontario, Canada; Medical University of South Carolina, UNITED STATES

## Abstract

**Background:**

We set out to determine the accuracy of 3D-navigated mandibular and maxillary osteotomies with the ultimate aim to integrate virtual cutting guides and 3D-navigation into ablative and reconstructive head and neck surgery.

**Methods:**

Four surgeons (two attending, two clinical fellows) completed 224 unnavigated and 224 3D-navigated osteotomies on anatomical models according to preoperative 3D plans. The osteotomized bones were scanned and analyzed.

**Results:**

Median distance from the virtual plan was 2.1 mm unnavigated (IQR 2.6 mm, ≥3 mm in 33%) and 1.2 mm 3D-navigated (IQR 1.1 mm, ≥3 mm in 6%) (P<0.0001); median pitch was 4.5° unnavigated (IQR 7.1°) and 3.5° 3D-navigated (IQR 4.0°) (P<0.0001); median roll was 7.4° unnavigated (IQR 8.5°) and 2.6° 3D-navigated (IQR 3.8°) (P<0.0001).

**Conclusion:**

3D-rendering enables osteotomy navigation. 3 mm is an appropriate planning distance. The next steps are translating virtual cutting guides to free bone flap reconstruction and clinical use.

## Introduction

In the conventional approach, head and neck surgeons osteotomize the mandible or maxilla according to the clinical appearance and imaging, and reconstruct with a free bone flap using a pre-contoured plate and surgical trial-and-error to make small intraoperative modifications to best fit the free flap.[1] Patient-specific cutting guides have been advocated by some groups to improve the precision and speed of complex maxillomandibular ablative and reconstructive head and neck surgery.[1–4] These cutting guides are designed and fabricated in advance of the surgery by computer-aided design and computer-aided modeling (CAD-CAM).

We developed a three-dimensional (3D) optical navigation system using virtual cutting guides as an alternative to the physical in-hand cutting guides manufactured using CAD-CAM. Virtual cutting guides may have benefits over in-hand cutting guides such as lower cost, adaptability to a change the operative plan (such as with tumor enlargement between the planning and operative stages), or the potential for reduced bone exposure if there is no physical guide to be attached to the bone. The fusion of preoperative planning with intraoperative image-guidance for osteotomies in head and neck tumor resections, orthognathic surgery and maxillomandibular reconstruction was proposed by Bell *et al* and other authors to facilitate the accuracy of ablation and enhance functional outcomes.[5–9] Our method of 3D-navigation overcomes the impediment experienced in guiding osteotomies using the separate triplanar views (axial, coronal and sagittal) of the conventional 2D-navigation systems that other groups have published on to date. The pre-planning for either 3D-navigation or CAD-CAM takes about an hour. The 3D optical navigation system could be applied to existing navigation systems without the need for the production of commercial physical cutting guides.

The primary aim of the present study was to assess the accuracy and reproducibility of 3D virtually-planned osteotomies in mandible and maxilla models using a novel 3D-navigation system and a navigated reciprocating saw. The secondary aims were to assess the minimum distance from the margin that cuts should be planned to ensure clearance, the challenges of registration error, how to affix the reference marker with the mandible being mobile, and minimizing obstructions to the infrared camera. We designed a laboratory study to compare the distance, pitch, and roll between planned and actual osteotomies using a free-hand reciprocating saw with and without 3D-navigation. This is the first step to integrating 3D-navigation with virtual cutting guides in head and neck ablative and reconstructive surgery.

## Materials and methods

### 3D-navigation system

**Fig 1:**
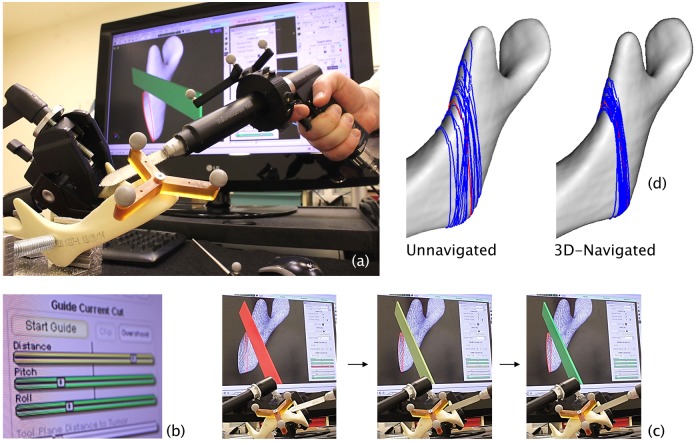
*GTx-Eyes* 3D-navigation system. (a), virtual cutting guide (red line) displayed on a CT reconstruction of the Sawbones mandible model, with navigated reciprocating saw correctly aligned (the saw blade is green) [bone anterior to the plane of the saw blade is clipped in the image so that the osteotomy plane can be visualized through the bone]; (b), the indicators of distance, pitch and roll move and change color from red to yellow then green as the navigated saw is aligned precisely with the virtual cutting guide; (c), the saw blade also turns from red to yellow then green as it is lined up correctly; (d), virtual cutting guide (red line) and unnavigated and 3D-navigated osteotomies (blue lines) after the analysis of multiple osteotomized models.

The present laboratory study utilized an in-house navigation system, *GTx-Eyes*, based on the open source Image-Guided Surgery Toolkit (Insight Software Consortium).[10, 11] The system uses powered saws tracked optically in real-time six degrees of freedom using the Polaris Spectra (NDI, Waterloo, ON, Canada) integrated with in-house 3D surface rendering, saw plane clipping, and colored accuracy indicators (Fig 1).

### Osteotomy planning

**Fig 2:**
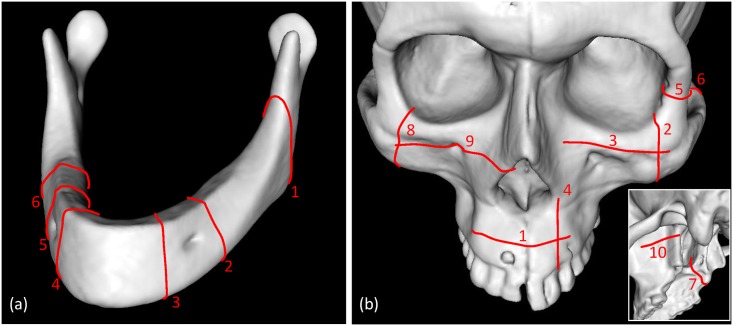
Planned osteotomies in Sawbones mandible and maxilla.

Data from a prototype cone-beam computed tomography (CT) system mounted on a mobile C-arm (PowerMobil, Siemens, Erlangen, Germany)[12] were acquired of a Sawbones (Vashon Island, WA, USA) mandible (model 1337–1) and skull (model 1344) (for the maxilla) and reconstructed in 3D with voxel size 0.8 mm^3^. A simulated ameloblastoma, osteosarcoma, and bone-invading squamous cell carcinoma based on past cases were drawn in the 3D CT reconstructions, and 1 cm resection margins were represented digitally around each simulated tumor. Corresponding ablative osteotomies were planned and drawn in 3D, totalling six osteotomies in the mandible model and ten osteotomies in the maxilla model (Fig 2). Sawbones manufactured the 48 mandibles and 12 skulls using the same two moulds and the same density of solid polyurethane. The mandibles were edentulous without teeth indentations, while the maxillae were dentate.

### Calibration and registration

The pointer and reciprocating saw, both with custom 3D-printed trackers and infrared-reflecting spheres, were calibrated using a custom planar jig. The reference marker, also with infrared-reflecting spheres, was attached with a cortical screw and washer to the symphysis menti of each Sawbones mandible and the mid-frontal bone of each Sawbones skull. The models were clamped to the workbench for convenience, although the reference markers permit the model to be mobile. The physical models were registered to the 3D reconstructions using four widely spaced fiducials in mandibles (the mental and mandibular foramina), and six widely spaced fiducials in the skulls (the upper third molars, the infraorbital foramina and the zygomaticofrontal sutures anteriorly). Image-to-tracker paired-point registration error was measured to assess how closely the preoperative CT and physical model were registered. Image-to-image registration error (the registration of the preoperative plan to the postoperative CT) was determined to confirm the accuracy of the quantitative outcome measures (distance, pitch, and roll).

### Osteotomies

Two attending surgeons and two clinical fellows performed the full series of osteotomies in two sittings each (mandibles followed by maxillae). The two attending surgeons had 25 and 3 years of attending experience. The Synthes (Solothurn, Switzerland) Battery Power Line II reciprocating saw and 68 mm x 1.1 mm Synthes blades were used for each osteotomy. The mandibular osteotomies were made subtotally (approximately 80% of the cut height) so that each bone remained intact and could be scanned whole to facilitate the analysis. In the skulls, the osteotomies were made fully because the skulls remained intact for scanning after the sequence of cuts had been completed. Each surgeon completed the unnavigated mandibular osteotomies using the 3D cut plans after first marking the cuts in pencil. Following a 15-minute training session with the navigation system, each surgeon completed the navigated mandibular osteotomies. The maxillae were then completed in the same sequence at a separate sitting without further navigation training.

The bone cuts were navigated using the 3D views by placing the saw on the start point of the cut, aligning the markers for distance, pitch, and roll and aligning the cut through the bone using surface clipping (Fig 1). The 3D view was usually oriented so as to look at the cut off-axis obliquely from above but this varied according to the preference of the surgeon undertaking the osteotomies, with the most experienced surgeon preferring to look at the osteotomy plane end-on. The bones were labelled by engraving with a 2 mm cutting burr.

### Osteotomy plane analysis

After completing the osteotomies, the mandibles and skulls were CT scanned with a SOMATOM Definition Flash (Siemens, Erlangen, Germany) and reconstructed in 3D with voxel size 0.9 x 0.9 x 0.6 mm. Each resection plane was defined in the 3D reconstructions by manually placing four evenly spaced digital points within each osteotomy and a plane of best fit was determined by random sample consensus (SRI International, Menlo Park, CA, USA). The CT reconstructions were then registered with the pre-operative CBCT scans in order to compare the osteotomies with the 3D virtual plans.

### Statistical analysis

The pre-operative virtual planned osteotomies and the actual osteotomies (unnavigated and navigated) were compared by determining the median in distance, pitch, and roll. Quantile-quantile plots of the variation in the distance, pitch, and roll of the osteotomies determined that the data were not normally distributed, so the Mann-Whitney U test was used for non-parametric comparisons of the deviation from the plans in distance, pitch, and roll in the unnavigated and navigated cuts. Interquartile ranges (IQR) were calculated to assist with the evaluation of accuracy and reproducibility. P-values <0.05 were deemed statistically significant. MATLAB (R2012a; MathWorks, Natick, MA, USA) was used for the statistical analyses.

## Results

**Fig 3:**
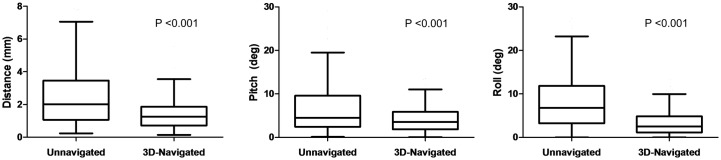
Box-and-whisker plots showing the distance, pitch and roll of the unnavigated and 3D-navigated osteotomies. Bars denote median, boxes show interquartile range (IQR), upper whiskers show third quartile plus 1.5 IQR, and lower whiskers show first quartile minus 1.5 IQR.

Four hundred and forty-eight osteotomies were made in total (S1 Supporting Information). Each of the four surgeons made 56 unnavigated and 56 3D-navigated osteotomies across 12 mandibles and four skulls. The navigated osteotomies were closer to the planned cut in distance, pitch, and roll than the unnavigated osteotomies (Table 1, Fig 3).

**Table 1:** Differences in distance, pitch and roll between the virtually planned osteotomy and the unnavigated and 3D-navigated osteotomies.

Site	Method (no. of osteotomies)	Distance (mm)					Pitch (deg)					Roll (deg)				
		Median	Q1	Q3	IQR	P	Median	Q1	Q3	IQR	P	Median	Q1	Q3	IQR	P
**All osteotomies**	Unnavigated (224)	2.1	1.1	3.7	2.6	<0.001	4.5	2.3	9.4	7.1	<0.001	7.4°	3.8	12.3	8.5	<0.001
	3D-Navigated(224)	1.2	0.7	1.8	1.1		3.5	1.8	5.8	4.0		2.6°	1.2	5.0	3.8	
**Mandible**	Unnavigated (144)	2.1	1.1	3.6	2.5	<0.001	3.9	2.1	6.9	4.8	0.36	7.3	4.4	11.5	7.1	<0.001
	3D-Navigated(144)	1.2	0.6	1.7	1.1		3.7	2.0	5.7	3.7		2.6	1.3	5.2	3.9	
**Maxilla**	Unnavigated(80)	2.1	1.2	3.8	2.6	<0.001	6.9	2.8	12.5	9.7	<0.001	7.8	3.0	13.9	10.9	<0.001
	3D-Navigated(80)	1.3	0.8	2.1	1.3		2.9	1.2	6.6	5.4		2.9	1.1	4.9	3.8	

Abbreviations: deg, degrees; IQR, interquartile range; P, P-value; Q1, first quartile upper boundary; Q3, third quartile upper boundary.

P-values were calculated by the Mann-Whitney U test.

Of the 224 osteotomies made without navigation, 115 (51%) diverged from the plan by ≥2.0 mm, 73 (33%) diverged by ≥3.0 mm, and 31 (14%) diverged by ≥5 mm from the planned cut. Of the 224 3D-navigated osteotomies, 48 (21%) diverged from the plan by ≥2.0 mm, only 14 (6%) diverged by ≥3 mm, and 2 (<1%) diverged by ≥5 mm (actually 5.5 cm and 6.6 cm) from the planned cut.

In mandibles and maxillae combined, the median distance from the plan in unnavigated osteotomies was 2.0 mm (IQR 2.6 mm) and in navigated osteotomies was 1.2 mm (IQR 1.1 mm) (P<0.001). The median difference in pitch from the plan was 4.5° (IQR 7.1°) unnavigated and 3.5° (IQR 4.0°) navigated (P<0.001). The median difference in roll from the plan was 7.4° (IQR 8.5°) unnavigated and 2.6° (IQR 3.8°) navigated (P<0.001). The combined and separate data for mandibles and maxillae are given in Table 1.

The median image-to-tracker paired-point registration error was 1.5 mm (IQR 0.4 mm) in mandibles and 1.2 mm (IQR 0.3 mm) in maxillae. The median image-to-image registration error was 1.4 mm (IQR 0.8 mm).

Attending surgeons were more accurate than clinical fellows with regard to distance and roll but not pitch of unnavigated osteotomies (Table 2). With navigation, there was no statistically significant difference between attending surgeons and clinical fellows (but the group sizes of two groups of two surgeons were small).

**Table 2:** Differences in median distance, pitch and roll between the virtually planned osteotomy and the unnavigated and 3D-navigated osteotomies of attending surgeons and clinical fellows.

Method	Clinical fellow/Attending surgeon (no. of osteotomies)	Distance (mm)					Pitch (deg)					Roll (deg)				
		Median	Q1	Q3	IQR	P	Median	Q1	Q3	IQR	P	Median	Q1	Q3	IQR	P
**Unnavigated**	Fellows (124)	2.9	1.7	4.9	3.2	<0.05	4.8	2.2	10.1	7.9	0.32	10.0	5.7	13.7	8.0	<0.05
	Attending (124)	1.4	0.9	2.3	1.4		4.3	2.5	7.5	5.0		6.1	3.0	10.4	7.4	
**3D-Navigated**	Fellows (124)	1.3	0.7	2.0	1.3	0.35	3.2	1.7	5.3	3.6	0.10	2.4	1.1	4.9	3.8	0.52
	Attending (124)	1.1	0.7	1.7	1.0		3.9	1.9	6.6	4.7		2.9	1.4	5.1	3.7	

Abbreviations: deg, degrees; IQR, interquartile range; P, P-value; Q1, first quartile upper boundary; Q3, third quartile upper boundary.

P-values were calculated by the Mann-Whitney U test.

The median values and IQRs were smaller in navigated osteotomies in all of the comparisons given in Tables 1 and 2, indicating improved accuracy and repeatability with 3D-navigation.

## Discussion

This study shows that optical 3D-navigation can ensure that greater than 99% of osteotomies are made with an accuracy of less than 5 mm of the computerized virtual surgical plan, compared with 86% without the navigation system. Without navigation, only 67% of cuts were within 3 mm and 49% were within 2 mm. With 3D-navigation, 86% of cuts were within 3 mm and 79% were within 2 mm. The median difference between the cut plan and all 3D-navigated osteotomies was 1.2 mm (IQR 1.1 mm), which compares favourably with mandibular osteotomies made with CAD-CAM physical cutting guides where the difference is 2.0 mm ± 1.1 mm.[1, 4]

3D-navigation is accurate in the preclinical setting for guiding saw planes and is more accurate than unnavigated cutting when executing a computerized virtual surgical plan. The distance from the intended resection margin that 3D-navigated osteotomies should be planned using the present navigation system is certainly within 5 mm and may be a distance of 3 mm. For osteotomies virtually planned at a distance of 10 mm from the edge of the bone tumour, the present navigation system would produce a negative (5 mm) bone margin in greater than 99% of cases.

Previous studies of intraoperative navigation in head and neck surgery have used 2D systems displaying separate triplanar views.[5–7, 9] 2D systems displaying triplanar views are only well-suited to tracking pointers and burrs to anatomical locations. It is difficult to align a saw to a plane using 2D systems, and osteotomies are only rarely parallel to the sagittal, coronal or axial planes.

In our 3D system of navigation, surface rendering of the 3D-reconstructed CT scan and surface clipping (virtual removal) of the bone to one side of the saw plane allows the surgeon to see through the image of the bone and align the plane of the saw blade with the cut plan. This allows the surgeon to judge the plane of the cut in distance, pitch, and roll and obtain a negative margin from the closest edge of the tumor within the bone.

The target registration error of commercially available triplanar otolaryngological surgical navigation systems varies between a mean of 1.3 mm (± 0.9 mm) and 2.8 mm (± 1.6 mm).[13] With the 3D-navigation system used for making osteotomies in the present study, the median distance of the plane from the plan was comparable at 1.5 mm (IQR 0.4 mm) in mandibles and 1.2 mm (IQR 0.3 mm) in maxillae. The slightly higher values for the mandible models may have arisen from subtle deformation during clamping and cutting because the mandibles had some flexibility compared to the skull models.

Without 3D-navigation, there was a statistically significant difference with regard to the accuracy of distance and roll but not pitch of unnavigated osteotomies between attending surgeons and clinical fellows which favored attending surgeons. But with navigation, there was no statistically significant difference between attending surgeons and clinical fellows which may indicate that navigation improves the performance of trainees. Further research is required to investigate this finding.

Accurate synchronization of the CT data with the mandible, which is mobile during surgery, is a challenge.[5] One potential solution is to place the patient into intermaxillary fixation with arch bars for the preoperative CT scan and navigated surgery, but this would prevent transoral access. Another option would be placing the jaw into centric relation manually, but this method is prone to movement and loss of registration.[5] Affixing a reference marker to the symphysis menti has proven successful with the present system with mandibular osteotomies, because the system can track mobile structures so long as the reference marker is fixed to that structure. In practice, this would be achieved by affixing the reference marker to a custom-printed centimeter-long post entering a puncture in the soft tissue envelope.

We anticipated problems from obstructions such as the surgeon between the infrared camera and the navigated saw and reference marker on the bone; this was prevented by mounting the camera vertically rather than obliquely above the field. An infrared camera integrated into overhead operative lighting and cameras is in early development. Blood on the reflective spheres on the reference marker and saw could be limiting in the clinical environment. Electromagnetic navigation would not be obstructed, but this mode of navigation has potential to be inaccurate with larger metallic instruments such as the saw.[14]

The reciprocating saw is started prior to bony contact to prevent a jump on the surface of the bone, but when using navigation the surgeons had a tendency to line up the saw with the virtual cutting guide and to place the saw in contact with the bone before starting the saw. The navigation system cannot account for flexion of the saw blade, so care must be taken to avoid flexion when lining up the saw blade with the planned osteotomy plane. The next stage will be to evaluate the system in the clinical environment.

The facility in head and neck surgical tumor ablation is relevant to the resection of osteogenic tumors such as ameloblastoma and osteosarcoma. Other potential areas of utility in ablative surgery include navigating the blind posterior osteotomies in maxillectomy, or sagittal split osteotomy and rim resection in mandibulectomy. A next preclinical step will be the evaluation of bony positive margins with and without 3D-navigation using anatomic models with visible tumor and intracortical erosion with the visible component of the tumor being the ‘tip of the iceberg’. The 3D-navigation system also allows features including superimposition of the next osteotomy on to the virtual saw blade to prevent crossing or overlap of the bone cuts, and proximity alerts[15]. With intraoperative cone-beam CT, the navigated image can be updated in near real time during resection and reconstruction.[12]

Future studies will compare 3D-navigated virtual cutting guides with physical in-hand cutting guides, and assess this technology for 3D-navigation of fibula free flap osteotomies in conjunction with the ablative osteotomies. This may be an alternative to using solid models and physical cutting guides in the CAD-CAM approach. Furthermore, the navigation system can track and record the osteotomy planes that have been completed, so it may be possible to have the computer automate the optimum digital reconstruction and corresponding virtual cutting guides for the fibula bone flap during the case.

## Conclusions

The distance, pitch, and roll of navigated mandibular and maxillary osteotomies were accurate to 2 mm and 3° in most cases, which was an improvement on unnavigated cuts. The range of values indicate the present 3D-navigation system will be sound for executing resection margins of 10 mm. The addition of 3D surface rendering to standard triplanar views enhances the utility of navigation for this application. Fixing a reference marker to the symphysis menti eliminates concerns about navigating osteotomies on the mobile mandible. The next steps will be to develop virtual cutting guides for osteotomized fibula free flaps and then translate and evaluate the combined ablation-reconstruction workstation in the operating room.

## Supporting information

S1 Supporting Information Minimal dataset. (XLSX)
